# Assessing the accuracy of ultrasound measurements of tracheal diameter: an in vitro experimental study

**DOI:** 10.1186/s12871-021-01398-3

**Published:** 2021-06-24

**Authors:** Ran Ye, Feifei Cai, Chengnan Guo, Xiaocheng Zhang, Dan Yan, Chengshui Chen, Bin Chen

**Affiliations:** 1 Department of Ultrasonography, The second Affiliated Hospital and Yuying children’s Hospital of Wenzhou Medical University, Wenzhou, 325006 Zhejiang China; 2 Department of Ultrasonography, Lucheng People’s Hospital of Wenzhou, Wenzhou, 325006 Zhejiang China; 3 Department of Preventive Medicine, School of Public Health & Management, Wenzhou Medical University, Wenzhou, 325006 Zhejiang China; 4 Key Laboratory of Interventional Pulmonology of Zhejiang Province, The First Affiliated Hospital of Wenzhou Medical University, Wenzhou, 325006 Zhejiang China; 5 Department of Pulmonary and Critical Care Medicine, Jinhua Municipal Central Hospital, Jinhua, 321000 Zhejiang China; 6 Department of Pulmonary and Critical Care Medicine, The First Affiliated Hospital of Wenzhou Medical University, Wenzhou, 325006 Zhejiang China; 7 Department of Ultrasonography, The First Affiliated Hospital of Wenzhou Medical University, Wenzhou, 325006 Zhejiang China

**Keywords:** Ultrasound, Endotracheal tube, Tracheal diameter, Tracheal wall pressure, Cuff inflation volume

## Abstract

**Background:**

Recent studies indicate that ultrasound can detect changes in tracheal diameter during endotracheal tube (ETT) cuff inflation. We sought to assess the accuracy of ultrasound measurement of tracheal diameter, and to determine the relationship between tracheal wall pressure (TWP), cuff inflation volume (CIV), and the degree of tracheal deformation.

**Methods:**

Our study comprised two parts: the first included 45 porcine tracheas, the second 41 porcine tracheas. Each trachea was intubated with a cuffed ETT, which was connected to an injector and the manometer via a three-way tap. The cuff was inflated and the cuff pressure recorded before and after intubation. The tracheal diameter was measured using ultrasound. This included three separate measurements: outer transverse diameter (OTD), internal transverse diameter (ITD), and anterior tracheal wall thicknesses (ATWT). A precision electronic Vernier caliper was also used to measure tracheal diameter. We calculated TWP and the percentage change of tracheal diameter. The Bland–Altman method, linear regression, and locally weighted regression (LOESS) were used to analyze the data.

**Results:**

There were strong correlation and agreement for OTD (*r* = 0.97, *P* < 0.001) and ITD (*r* = 0.90, *P* < 0.001) as measured by ultrasound and by precision electronic Vernier caliper, but a poor correlation for ATWT (*r* = 0.58, *P* < 0.001). There was a strong correlation between the percentage change of OTD (OTD%, *r* = 0.75, *P* < 0.001) and CIV, the percentage change of ITD (ITD%, *r* = 0.77, *P* < 0.001) and CIV, TWP (*r* = 0.75, *P* < 0.001) and CIV. And a strong correlation was also found between TWP and OTD% (*r* = 0.84, *P* < 0.001), TWP and ITD% (*r* = 0.84, *P* < 0.001).

**Conclusions:**

Use of ultrasound to measure OTD and ITD is accurate, but is less accurate for ATWT. There is a close correlation between OTD%, ITD%, CIV and TWP.

**Supplementary Information:**

The online version contains supplementary material available at 10.1186/s12871-021-01398-3.

## Background

Airway ultrasound can provide detailed images of the upper airway, including the thyroid cartilage, vocal cords, and trachea [1, 2]. Ultrasound has been shown to detect accurately the position [3–5] and depth of endotracheal tube (ETT) [6, 7] during intubation. The tracheal diameter measured by ultrasound is the basis of several studies. Certain studies [8, 9] indicated that ultrasound measurement of tracheal diameter is reliable. An animal study measured the tracheal diameter to assess tracheal collapse [10]. Clinical studies demonstrate that laryngeal ultrasonography can measure width difference of the air column before and after deflation of endotracheal tube cuff, which may be a predictor of post-extubation stridor [11, 12].

To the best of our knowledge, the accuracy and veracity of ultrasound measurements can depend on the experience of the sonographer. Stuntz [13] and Gottlieb [14] both observe that, when compared with the clinician, the professional sonographers obtain better airway ultrasound images and interpret images with greater accuracy and acuity. Chou [15] indicates that with proper training, clinicians can undertake airway ultrasonography and obtain accurate and reliable results. Julio [16] trained three second-year anesthesiology residents, showing tracheal internal transverse diameter measurements obtained by different operators were both reliable and precise.

Endotracheal intubation is often performed in patients with respiratory failure. It maintains airway patency by establishing artificial airways, and supports subsequent mechanical ventilation. The cuff is inflated with air to create a seal within the airway. This helps maintain positive pressure ventilation and prevents micro-aspiration of fluid secretion. Many studies recommend cuff pressure is monitored and kept between 20 and 30 cmH_2_O [17]. Tracheal wall pressure (TWP) is the pressure that endotracheal cuff exerts on tracheal wall. Despite the high-volume, low-pressure cuff pressure is related to tracheal wall pressure, but it is not exactly the same. If tracheal wall pressure does not exceed the capillary pressure of tracheal mucosa, complications arising from intubation are reduced [18]. Ramsingh *el at.* showed that inflation of endotracheal tube cuff increases trachea diameter, which can be observed using ultrasound [6, 19]. As tracheal wall pressure and tracheal deformation are caused by inflation of endotracheal tube cuff, a correlation is possible between tracheal wall pressure, cuff inflation volume (CIV), and the degree of tracheal deformation as determined by ultrasound.

In this study, our primary aim was to assess the accuracy of ultrasound measurements of the three tracheal diameters. Our second aim was to explore the relationship between tracheal wall pressure, cuff inflation volume, and the degree of tracheal deformation as measured by ultrasound.

## Methods

### Materials

All animal studies were conducted under the oversight of the Institutional Animal Care and Use Committee of Wenzhou Medical University (Wenzhou, China). In the present study, 45 porcine tracheas were obtained from animals sacrificed within 24 h in local abattoirs. No living animals were used in this study. Each tracheal specimen consisted of the upper larynx, trachea, and part of the right and left main stem bronchus.

An ultrasonography device (EZU-MT28-S1, HITACHI, Japan, Tokyo) with a 5–13 Hz linear probe was used for ultrasonography. The balloon of ETT was connected to a 10 mL injector and a digital manometer (PLD.0201, BOOST, China) through a three-way tap. The range of the digital manometer is 0–35 kPa and the accuracy 0.2%.

As the anterior cervical tissue was not present in the porcine trachea, ultrasound images were affected by the presence of air. We prepared a thin-walled approximately 150 mm long water bladder by loading 100 mL water into a condom. The water bladder was placed perpendicular to the long axis of the trachea. The water bladder was light and soft, and was used as acoustic window to allow proper display of resulting tracheal images [20]. Tracheal structure remained mostly unchanged. Ultrasonography gel was applied between the tracheal surface and the water bladder.

The trachea was positioned so that it lay flat supported on a rigid bracket on the table. It was then intubated with an 8.0 mm oral/nasal tracheal tube (Covidien, USA, Mansfield). The outer diameter of the inflation cuff is 27 mm. An ETT was placed at a depth of 18 mm, based on the distance from the thyroid cartilage. Tape was used to secure the porcine trachea to the rigid bracket, ensuring a constant position during measurement. A transverse line was drawn on the trachea to mark the center of the cuff.

### Ultrasonographical features of the porcine trachea

The porcine trachea was semicircular in the transverse plane, resembling an inverted U. The cartilage of the tracheal rings was hypoechoic; if calcification occurs, it may become hyperechoic. The outer edge of the trachea presented a hyperechoic strip with a clear smooth boundary. The inner surface of the trachea was linearly hyperechoic, and is known as the air-mucosal interface (A-M interface). The posterior part of the trachea was the reverberation artifact.

**Fig. 1:**
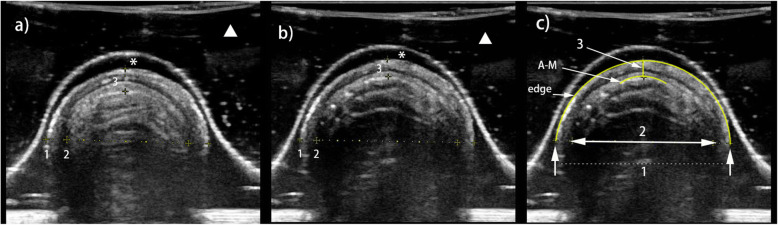
The sonogram shows the porcine trachea in the transverse plane. Water bladder (▲); ultrasonography gel (*); lines indicate outer transverse diameter (OTD) (1), internal transverse diameter (ITD) (2) and anterior tracheal wall thicknesses (ATWT) (3). **a** Cuff inflation volume is 0 mL. **b** Cuff inflation volume is 10 mL. **c** Tracheal diameter measurement. OTD (width between two white arrows; dotted line); ITD (two white arrowheads); ATWT (yellow vertical line); A-M interface (A-M); tracheal outer edge

The outer transverse diameter (OTD) was defined as the distance between the hyperechoic regions on both sides of the tracheal edge. The internal transverse diameter (ITD) was defined as the distance between the A-M interfaces of both sides. The ITD showed a hypoechoic edge on the ultrasound. The anterior tracheal wall thickness (ATWT) was defined as the distance from the hyperechoic front wall to the A-M interface (Fig. 1).

### Pressure difference technique

The TWP was estimated using the following formula [21]:

$$ \mathrm{TWP}={\mathrm{CP}}_{\mathrm{inserted}}-{\mathrm{CP}}_{\mathrm{uninserted}} $$

The uninserted cuff pressure (CP_uninserted_) is a ETT cuff pressure measured after inflating with a set volume of air in vitro. The inserted cuff pressure (CP_inserted_) is the pressure generated after the ETT intubated into the trachea and the cuff inflated with the same volume of air. In the same ETT, the pressure generated after every 1 mL increment of inflation with air was measured using the digital manometer.

### Study process

A researcher injected air into the uninserted ETT cuff (with an incremental increase of 1 mL) through a three-way tap while recording CP_uninserted_ and CIV. After the ETT was inserted into the porcine trachea, the same researcher repeated this procedure, recording CP_inserted_. A professional sonographer used a 5–13 Hz linear probe to acquire the transverse plane image of each CIV. Ultrasonography was performed directly above the marker line, with the probe perpendicular to the table. Measurements were taken until CIV reached 10 mL. All measurements were repeated three times. A new ETT was used for each porcine trachea.

OTD, ITD, and ATWT were measured by the sonographer. The percentage change of OTD (OTD%) was calculated using the following formula:

$$ \mathrm{OTD}\%=\left(\mathrm{OTD}-\mathrm{OTD}0\right)/\mathrm{OTD}0\times 100 $$

The OTD was measured for each CIV. OTD0 was the OTD of the trachea when the CIV was 0 mL. The percentage change of ITD (ITD%) was calculated similarly. The percentage change of ATWT (ATWT%) was calculated using the following formula:

$$ \mathrm{ATWT}\%=\left(\mathrm{ATWT}0-\mathrm{ATWT}\right)/\mathrm{ATWT}0\times 100. $$

When CP_uninserted_, CP_inserted_, OTD, ITD, and ATWT of a given trachea were measured, a precision electronic Vernier caliper was used to measure the OTD along the marked line after removal of the ETT. A cross transverse incision was made through the trachea to measure the ITD and ATWT. Each diameter was assessed three times by a researcher.

### Statistical analysis

Statistical analyses were conducted using MedCalc19.0.4 and SAS9.4. The distribution of continuous variables was analyzed using the Kolmogrov–Smirnov test. Normally distributed variables were summarized as mean and standard deviation. Non-normally distributed variables were presented as median and by the interquartile range. The Bland–Altman method and linear regression were used to assess the accuracy of and the agreement between measurements made using the precision electronic Vernier caliper and by ultrasound. Locally weighted regression (LOESS) was used to observe changes in TWP, CIV and the percentage change of tracheal diameter. Pearson correlation analysis was used to assess normally distributed variables and Spearman correlation analysis was used to assess non-normally distributed variables. A *P* value < 0.05 was considered significant.

## Results

### Analysis of the agreement and accuracy in the measurement of the tracheal diameter using ultrasound

The first part included 45 porcine tracheas. Sample size calculation and power analysis were performed (Supplementary Figure 1, 2 and 3). Table 1 shows the measurements of the tracheal diameter using both ultrasound and the precision electronic Vernier caliper.

**Table 1:** The tracheal diameter measured by precision electronic Vernier caliper and ultrasound, Mean ± SD (mm)

Tracheal diameter	Precision electronic Vernier caliper	Ultrasound
OTD	26.64 ± 2.12	26.83 ± 2.00
ITD	21.59 ± 1.71	21.26 ± 2.04
ATWT	3.64 ± 0.57	3.42 ± 0.57

*OTD* Outer transverse diameter, *ITD* Internal transverse diameter, *ATWT* Anterior tracheal wall thicknesses

**Fig. 2:**
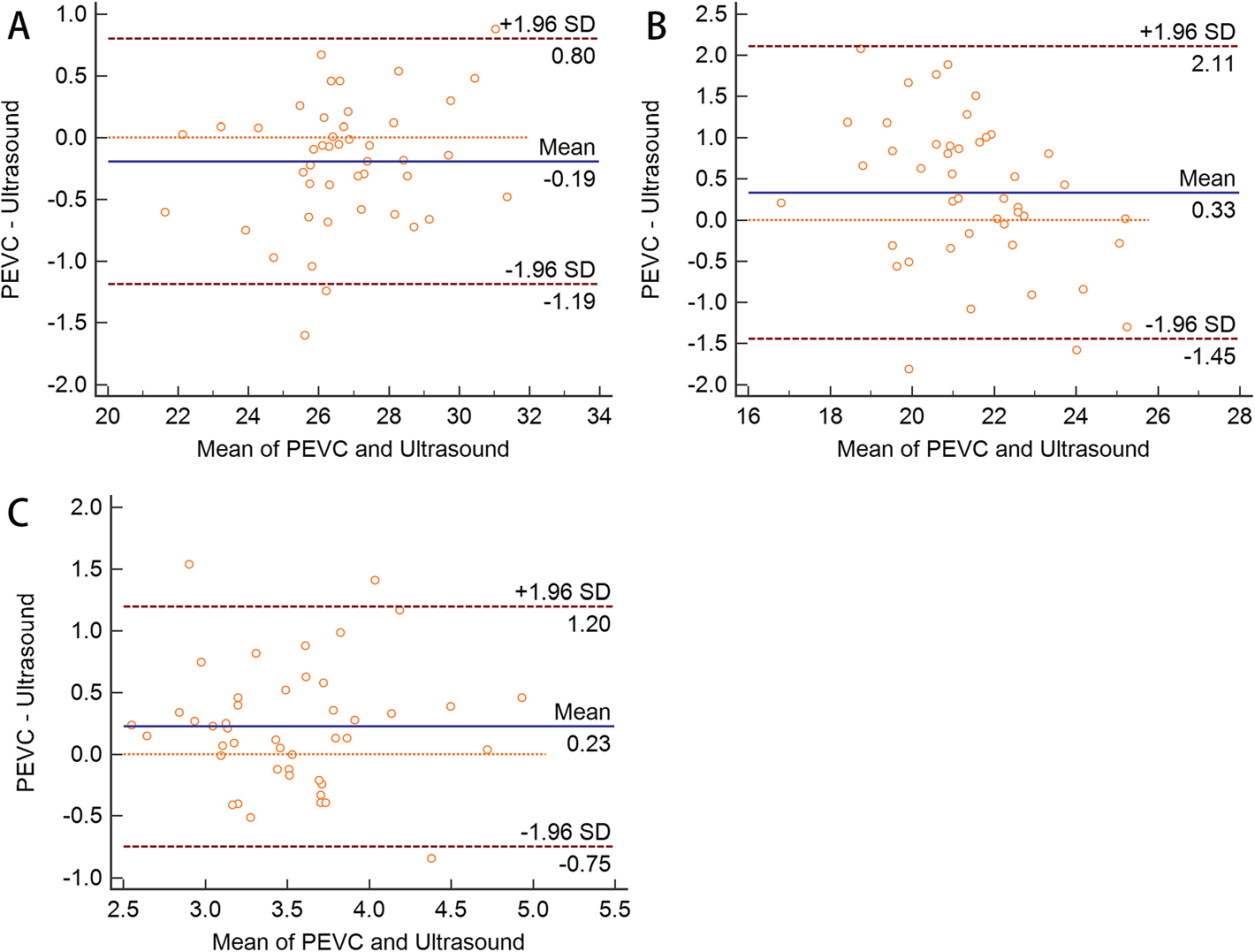
Bland–Altman analysis of the precision electronic Vernier caliper (PEVC) and ultrasound measurements of tracheal outer transverse diameter (**A**). Internal transverse diameter (**B**). Anterior tracheal wall thicknesses (**C**). The solid line indicates the bias (average difference between paired measurements) and the dotted lines indicate limits of agreement (1.96 ± SD)

Bland–Altman analysis and linear regression, indicated a strong correlation between precision electronic Vernier caliper and ultrasonography measurements of OTD (*n* = 45, *r* = 0.97, *P* < 0.001). We noted a bias of − 0.19 mm with a precision of 0.08 mm. The limit of agreement was − 1.19/0.80 mm (Fig. 2A). With respect to ITD, there was also a strong correlation between the methods (*n* = 45, *r* = 0.90, *P* < 0.001). We observed a bias of 0.33 mm with a precision of 0.14 mm. The limit of agreement was − 1.45/2.11 mm (Fig. 2B).

For ATWT, there was a poor correlation between the methods (*n* = 45, Spearman’s rank correlation coefficient: 0.58, *P* < 0.001). We noted a bias of 0.23 mm with a precision of 0.07 mm. The limit of agreement was − 0.75/1.20 mm (Fig. 2C).

### The relationship between the tracheal diameter, tracheal wall pressure, and cuff inflation volume

The second part included 41 porcine tracheas. Sample size calculation and power analysis were performed (Supplementary Figure 4). Four tracheas were excluded because of their excessively large inner diameter, which was larger than the maximum outer diameter of the cuff after inflation. Four hundred fifty-one sets of measurements were recorded (Table 2). As the ATWT correlation between the methods was poor, the resulting measurements were deemed to be inaccurate. Thus, subsequent analysis was suspended.

**Table 2:** Median (IQR) of TWP, OTD%, and ITD% for different cuff inflation volume

CIV (mL)	TWP (mmHg)	OTD%	ITD%
0	0 (0, 0)	0.00 (0.00, 0.00)	0.00 (0.00, 0.00)
1	1 (1,1)	0.24 (−0.12, 0.36)	0.54 (0.00, 1.20)
2	1 (1, 1)	0.25 (0.00, 0.50)	0.65 (0.00, 1.82)
3	1 (1, 2)	0.26 (0.00, 0.51)	1.26 (0.15, 1.88)
4	2 (1, 3)	0.37 (0.13, 1.03)	1.50 (0.81, 2.25)
5	4 (2, 6)	0.91 (0.35, 2.30)	2.35 (1.13, 3.17)
6	7 (3, 11)	1.67 (0.62, 3.62)	3.54 (2.05, 5.75)
7	12 (6, 17)	3.09 (1.49, 5.50)	5.61 (3.46, 8.70)
8	17 (11, 23)	4.34 (2.65, 7.25)	7.74 (4.65, 11.92)
9	23 (16, 31)	5.95 (4.35, 8.25)	9.70 (6.64, 14.14)
10	28 (20, 41)	7.55 (5.39, 9.12)	11.20 (8.16, 14.90)

*CIV* Cuff inflation volume, *TWP* Tracheal wall pressure, *OTD%* Percentage change of outer transverse diameter, *ITD%* Percentage change of internal transverse diameter

**Fig. 3:**
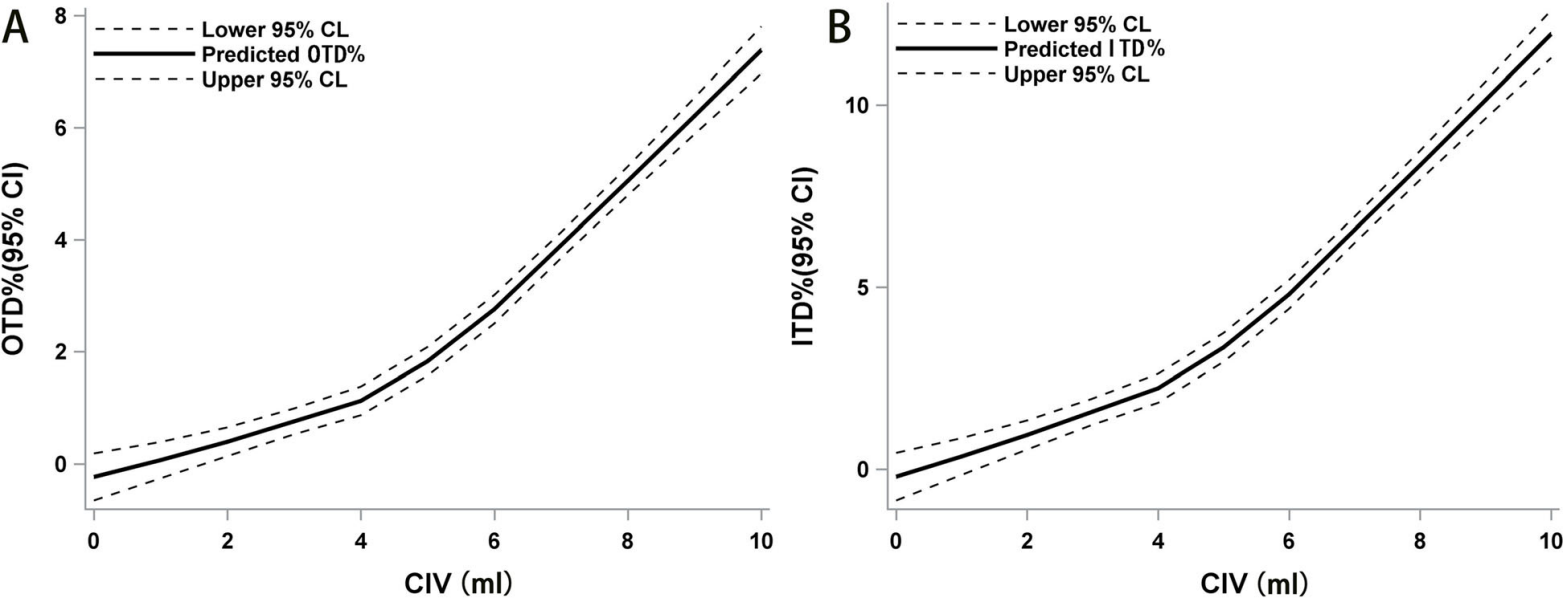
The relationship of the CIV, OTD%, and ITD% based on LOESS. The solid line indicates the trends. The dotted lines indicate the 95% confidence interval

Correlations between CIV, TWP, OTD%, and ITD% as measured by ultrasound, are shown in Table 3. LOESS showed that OTD% and ITD% were approximately linear with respect to the CIV, despite an inflection point at 4 ml (Fig. 3A-B). A strong correlation was observed between CIV and OTD% (*r* = 0.75), ITD% (Four hundred fifty-one = 0.77).

**Table 3:** Relationship of cuff inflation volume (CIV), tracheal wall pressure (TWP), OTD% and ITD%

	Pearson Correlation coefficient, *N* = 451			
	CIV	TWP	OTD%	ITD%
CIV	1.00	0.75 < 0.001	0.75 < 0.001	0.77 < 0.001
TWP	0.75 < 0.001	1.00	0.84 < 0.001	0.84 < 0.001
OTD%	0.75 < 0.001	0.84 < 0.001	1.00	0.95 < 0.001
ITD%	0.77 < 0.001	0.84 < 0.001	0.95 < 0.001	1.00

*CIV* Cuff inflation volume, *TWP* Tracheal wall pressure, *OTD%* Percentage change of outer transverse diameter, *ITD%* Percentage change of internal transverse diameter

**Fig. 4:**
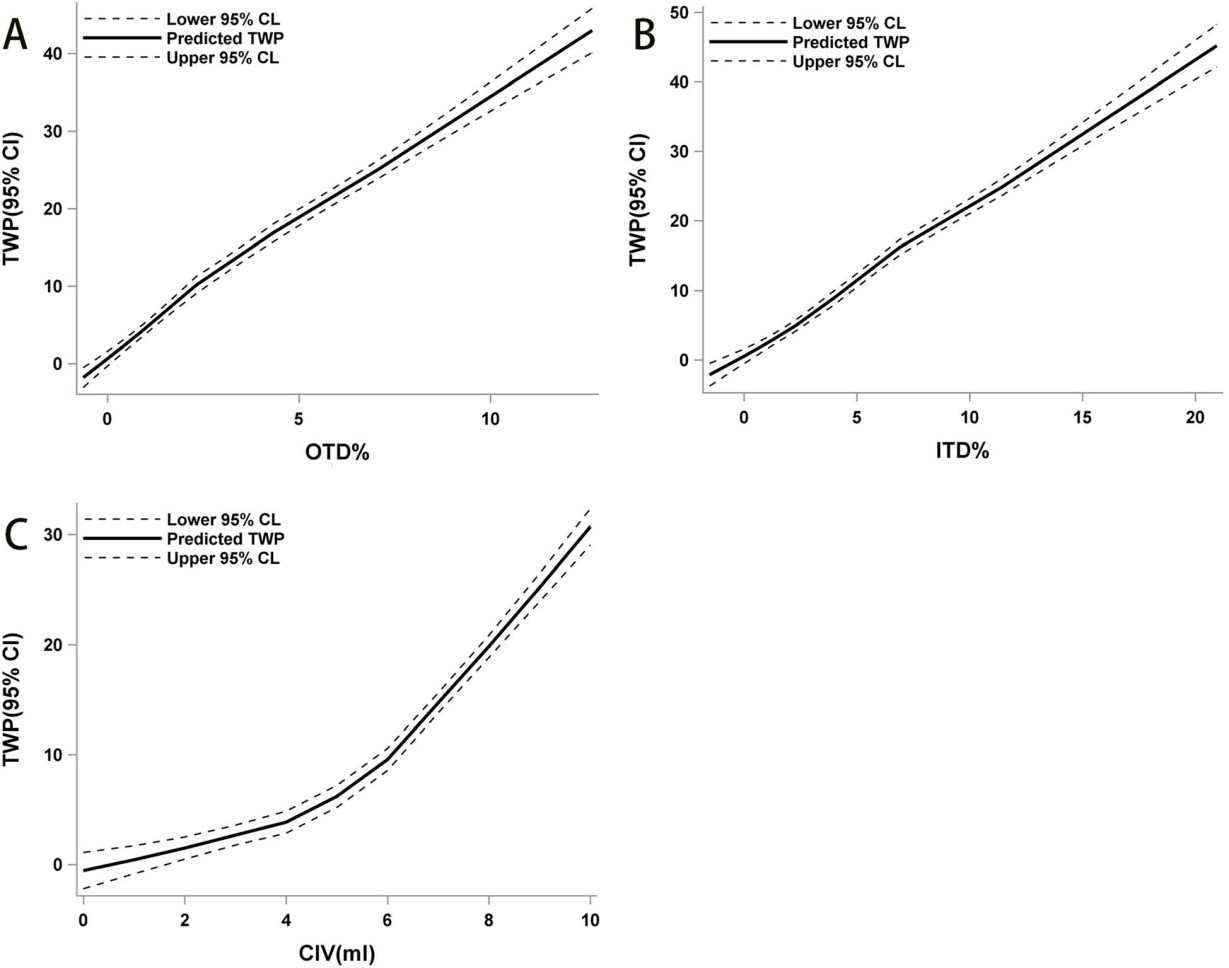
The relationship of TWP, OTD%, ITD%, and CIV based on LOESS. The solid line indicates the trends. The dotted lines indicate the 95% confidence interval

LOESS demonstrated that OTD% and ITD% were approximately linear with the TWP (Fig. 4A-B). A strong correlation was found between TWP and OTD% (*r* = 0.84), ITD% (*r* = 0.84).

The CIV showed a curvilinear relationship with TWP. When CIV was 0–4 mL, the TWP increased slowly. The TWP trend increased more when CIV was 4–6 mL. When CIV was 6–10 mL, TWP increased significantly with the increase in CIV (Fig. 4C). A strong correlation was found between CIV and TWP (*r* = 0.75).

## Discussion

Previous studies have shown that ultrasound can be a reliable tool for the assessment of tracheal diameter. In these studies, the tracheal outer transverse diameter (OTD) [9], internal transverse diameter (ITD) [8], and anterior tracheal wall thicknesses (ATWT) [22] were used as the principal ultrasound measurements, respectively. However, no studies have compared which diameter was measured more accurately. This experimental study assessed the accuracy of the ultrasound measurement of three separate tracheal diameters, and investigated the relationship of the percentage change of tracheal diameter, with tracheal wall pressure and cuff inflation volume.

Our results indicate that ultrasonography correlates strongly with the OTD and ITD measurements made by precision electronic Vernier caliper. And the limit of agreement for OTD was narrower. Therefore, ultrasound measurement of OTD was more accurate than measurement of ITD. The ATWT correlation between the two methods was poor, so the resulting ultrasound measurements were deemed inaccurate.

Julio [16] *el at.* studied the inter-rater and intra-rater reliability of ultrasound measurement of airway diameter. They showed that ultrasound measurement of ITD is both reliable and precise. Lakhal [8] *el at.* compared ultrasound and magnetic resonance imaging measurements of ITD. Sustic [9] et al. compared ultrasound and computed tomography measurements of OTD. In our study, there were strong correlation and agreement between the two methods when measuring the OTD and ITD. The results of our study agree closely with those of Lakhal *el at.* and Sustic *el at..* Shih [22] *el at.* proposed that anterior tracheal wall thicknesses can be measured at the thyroid isthmus level with ultrasound. This result contrasted with ours. We showed that the ultrasound measurement of ATWT was inaccurate. As Shih et al. only described the results of ultrasound measurements, without applying additional verification, the inaccuracy of ATWT measurement was not apparent. Moreover, the thin anterior wall of the trachea may pose a difficulty for ultrasound measurements.

The second part of our study found that OTD% and ITD% measured using ultrasound correlates strongly with cuff inflation volume and tracheal wall pressure.

A study of ovine trachea explored a universally optimal cuff inflation volume [23]. The results were less than satisfactory, and their results chosen range of cuff inflation volumes (5-7 ml) did not achieve a safe cuff pressure (20–30 cmH_2_O). A prospective Japanese study [24] used tracheal diameter from chest X-ray to evaluate the cuff inflation volume and compared it with an equation combining height and age. The results indicated that an equation based on tracheal diameter (Optimal cuff inflation volume = 0.71 (tracheal diameter) - 8.25, the adjusted coefficient of determination being 0.83) was better than the equation combining height and age (optimal cuff inflation volume = 0.11 (height) + 0.042 (age) - 15.6, the adjusted coefficient of determination being 0.44).

Some studies show that cuff inflation causes tracheal dilation, which could be observed using ultrasound on the suprasternal notch plane [6, 19]. Therefore, ultrasound can be used instead of X-ray to measure tracheal diameter. Compared with X-ray, ultrasound has the advantage of being fast, convenient, and non-invasive, providing real-time measurements. In our study, OTD% and ITD% correlated strongly with the cuff inflation volume. An ultrasound-guided cuff inflation protocol should also be explored. We will investigate this in our next study.

While the cuff inflation volume is responsible for tracheal sealing, tracheal wall pressure determines potential ischemia [18]. Tracheal wall pressure is exerted by endotracheal cuff on tracheal wall. The tracheal wall pressure and the cuff pressure are distinct concepts. Some studies report tracheal wall pressure was lower than the cuff pressure [25–27]. Techniques used to measure the tracheal wall pressure included the pressure difference technique, the wall pressure membrane technique, and the microchip sensor probe technique. The wall pressure membrane technique requires perforating the trachea wall and covering it with a membrane connected to an electronic transducer. It’s only suitable for in vitro studies [21]. The microchip sensor probe is known to generate artificially high pressures between cuff and trachea [28]. Both techniques are limited by the cost of acquisition and maintenance. In our study, we chose the pressure difference technique [29] for estimation of tracheal wall pressure as it is easy to use and provides relatively reliable results [21, 28]. Its principal disadvantage is that it can only assess the overall pressure of the tracheal wall. Brimacombe [28] *el at.* showed the cuff will cause different tracheal wall pressure at different sites during inflation. Tracheal wall pressure has received little attention in clinical practice, which may be the reason for the lack of measurement methods. The pressure difference technique can be a method, but it is still complicated. Our results indicate a strong correlation between tracheal wall pressure and OTD%, ITD%. We wish to explore further whether tracheal wall pressure can be estimated using the tracheal diameter difference as measured by ultrasound.

This study has certain limitations. Firstly, we performed our study using porcine trachea in vitro rather than human trachea in vivo. Although porcine tracheal diameter is generally larger than that of humans, it is similar in structure to the human trachea. In vitro, insufficient perfusion and temperature reduction in the extracorporeal trachea may reduce the elasticity of the tissue. Secondly, we chose the 8.0 mm oral/nasal tracheal tube by Covidien, which is in common use within clinical settings. However, endotracheal tubes have several manufacturers, leading to different cuff inflation volume, cuff diameters, and variable composition of the materials used for the endotracheal tube. Thus, our findings may not apply to other manufacturers or sizes of endotracheal tube.

## Conclusions

In conclusion, we showed that ultrasound measurements of OTD and ITD are reliable, and that the accuracy of ultrasound measurement of OTD is better than that of ITD. But the measurement of the ATWT is inaccurate. Additionally, OTD% and ITD%, as measured by ultrasound, correlates strongly with cuff inflation volume and tracheal wall pressure. Our study provides a basis for further development of airway ultrasound applications, such as ultrasound-guided cuff inflation protocol or using ultrasound to assess tracheal wall pressure.

## Supplementary Information

**Additional file 1: Figure S1.** The sample size estimated from the previous reference [8, 9] which their correlation coefficient is 0.882.

**Additional file 2: Figure S2.** In our study, we evaluated inversely whether the sample size was sufficient. The sample size estimated with the correlation coefficient is 0.9 (Form tracheal internal transverse diameter).

**Additional file 3: Figure S3.** The sample size estimated from our study, with the correlation coefficient is 0.58 (Form anterior tracheal wall thicknesses).

**Additional file 4: Figure S4.** The sample size estimated from our study, according to the minimum correlation coefficient (*r* = 0.75) in the second part results.

## Data Availability

The datasets used and/or analysed during the current study are available from the corresponding author on reasonable request.

## Abbreviation

ETT: Endotracheal tube
TWP: Tracheal wall pressure
CIV: Cuff inflation volume
OTD: Outer transverse diameter
OTD%: Percentage change of outer transverse diameter
ITD: Internal transverse diameter
ITD%: Percentage change of internal transverse diameter
ATWT: Anterior tracheal wall thicknesses
ATWT%: Percentage change of anterior tracheal wall thicknesses
CP_inserted_: Inserted cuff pressure
CP_uninserted_: Uninserted cuff pressure
