# Supplementary figures and images for: Assessing the accuracy of ultrasound measurements of tracheal diameter: an in vitro experimental study
Source: BMC Anesthesiol. 2021 Jun 24;21:177. doi: 10.1186/s12871-021-01398-3 (PMC8223278; doi:10.1186/s12871-021-01398-3)

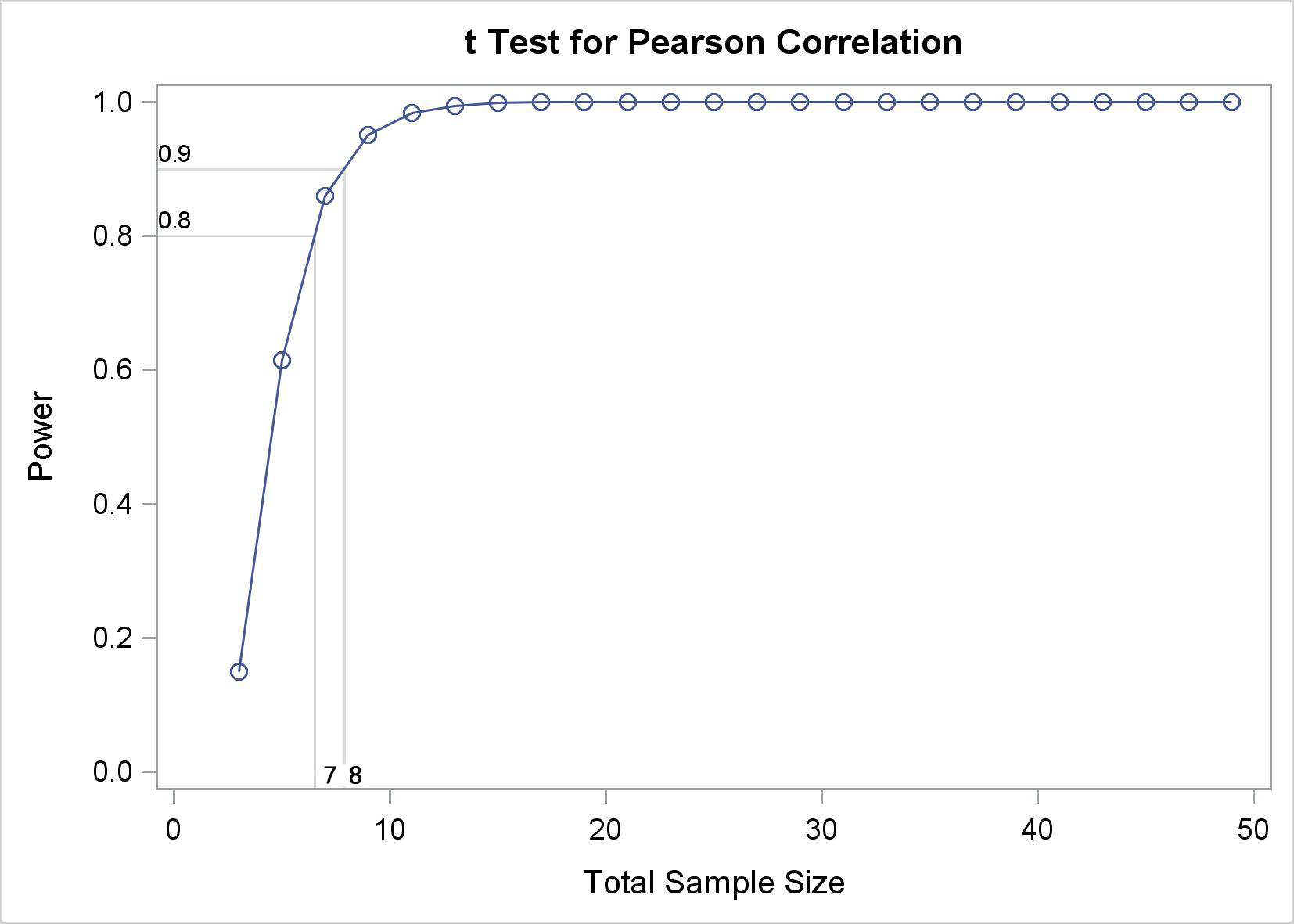

Supplement: Supplementary file 1 — Additional file 1: Figure S1. The sample size estimated from the previous reference [8, 9] which their correlation coefficient is 0.882. [file 12871_2021_1398_MOESM1_ESM.tif]

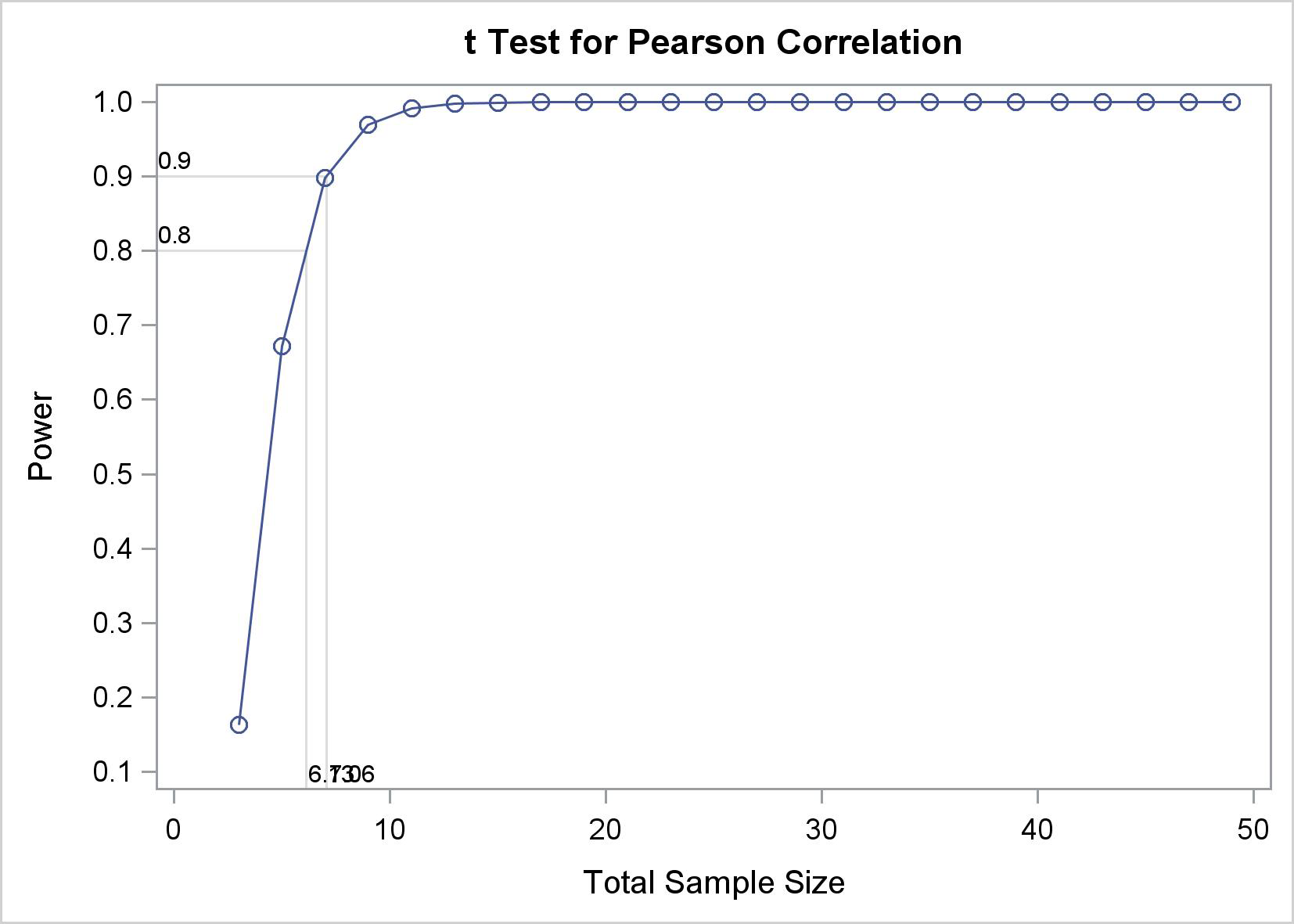

Supplement: Supplementary file 2 — Additional file 2: Figure S2. In our study, we evaluated inversely whether the sample size was sufficient. The sample size estimated with the correlation coefficient is 0.9 (Form tracheal internal transverse diameter). [file 12871_2021_1398_MOESM2_ESM.tif]

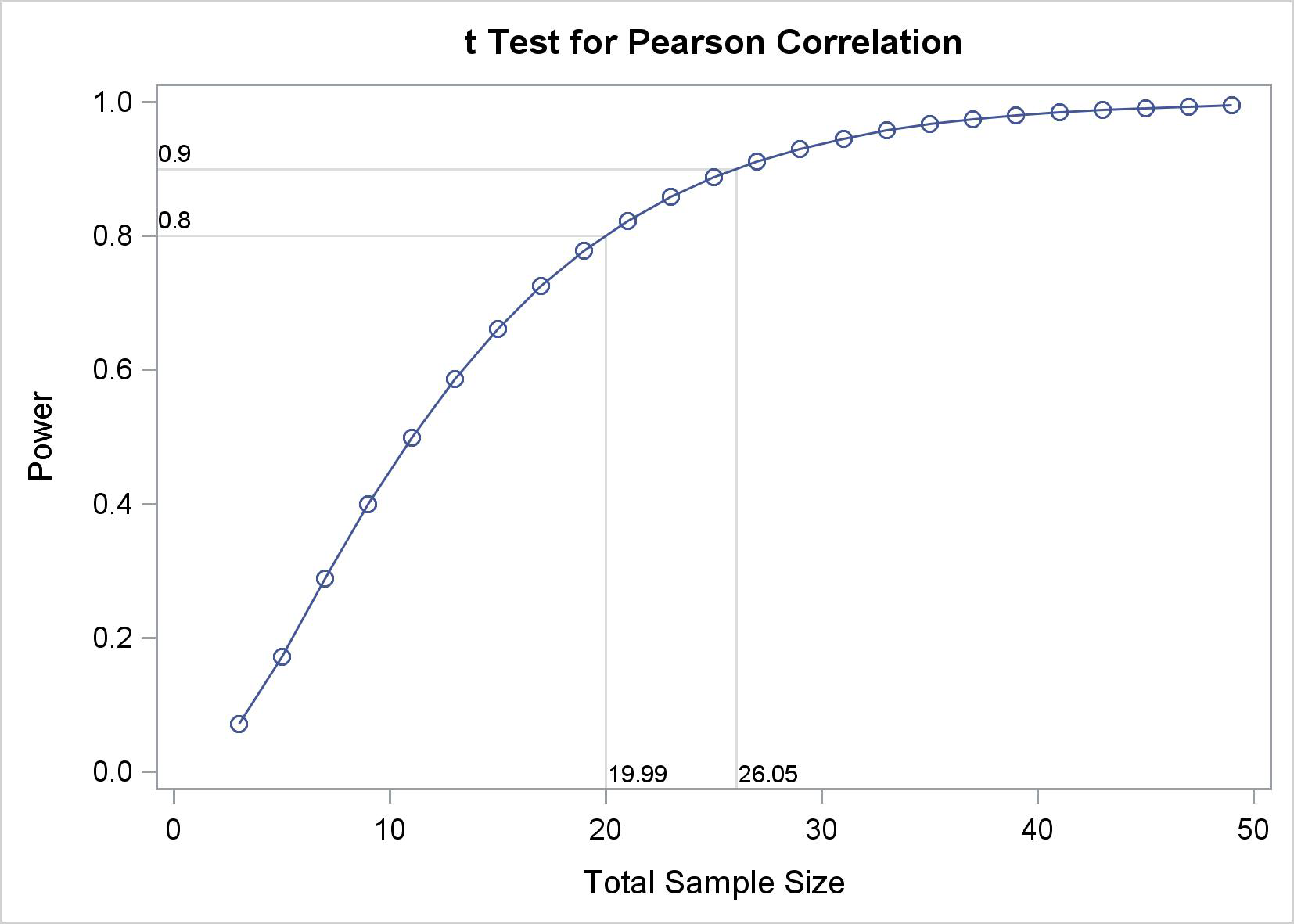

Supplement: Supplementary file 3 — Additional file 3: Figure S3. The sample size estimated from our study, with the correlation coefficient is 0.58 (Form anterior tracheal wall thicknesses). [file 12871_2021_1398_MOESM3_ESM.tif]

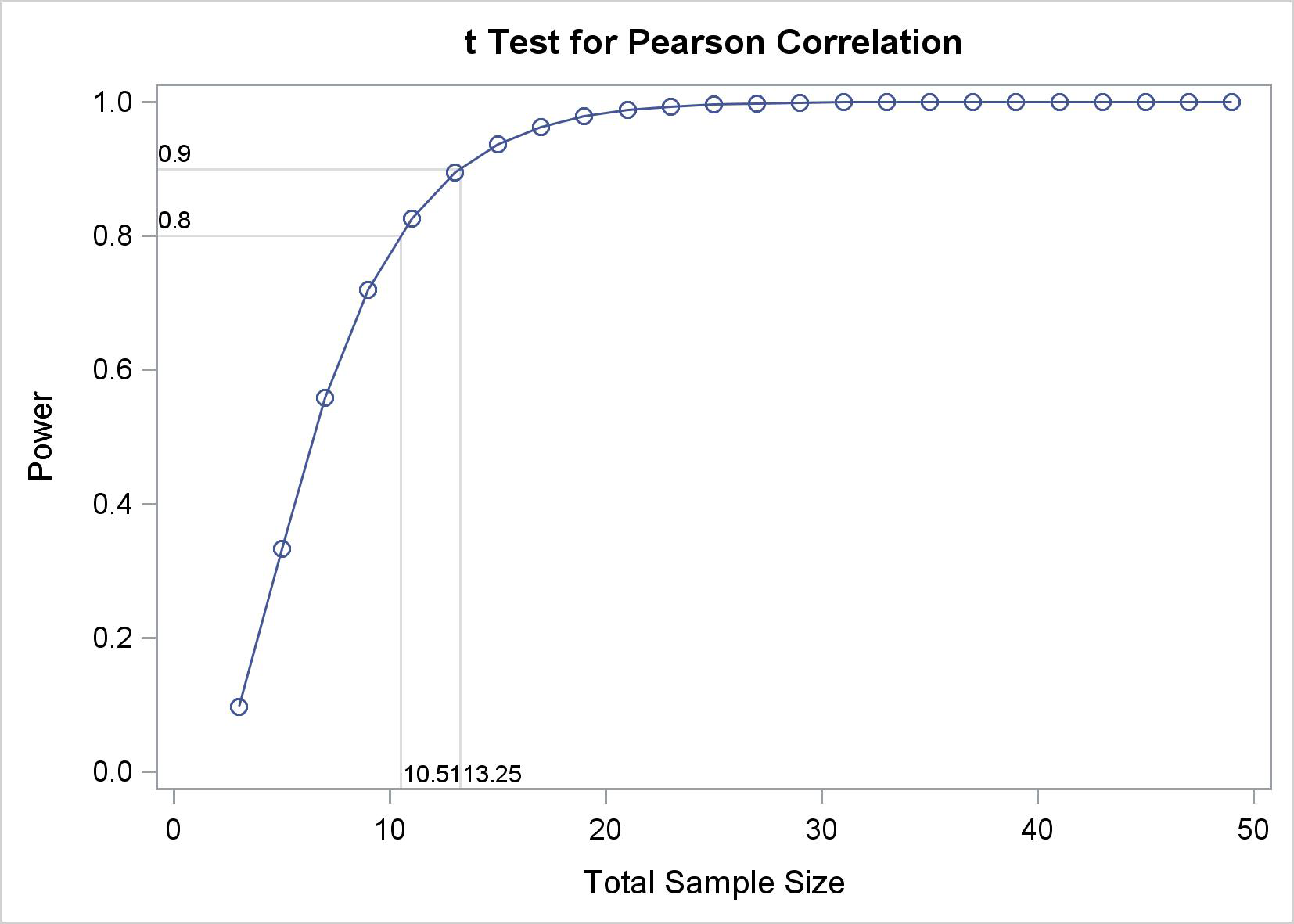

Supplement: Supplementary file 4 — Additional file 4: Figure S4. The sample size estimated from our study, according to the minimum correlation coefficient (r = 0.75) in the second part results. [file 12871_2021_1398_MOESM4_ESM.tif]
